# Recombinant tandem of pore-domains in a Weakly Inward rectifying K^+^ channel 2 (TWIK2) forms active lysosomal channels

**DOI:** 10.1038/s41598-017-00640-8

**Published:** 2017-04-05

**Authors:** Nicole Bobak, Sylvain Feliciangeli, Cheng-Chang Chen, Ismail Ben Soussia, Stefan Bittner, Sophie Pagnotta, Tobias Ruck, Martin Biel, Christian Wahl-Schott, Christian Grimm, Sven G. Meuth, Florian Lesage

**Affiliations:** 1 Université Côte d’Azur, Inserm, CNRS, Institut de Pharmacologie Moléculaire et Cellulaire, LabEx ICST, 660 Route des Lucioles, 06560 Valbonne, France; 2 Center for Integrated Protein Science CIPS-M and Department of Pharmacy - Center for Drug Research, Ludwig-Maximilians-Universität München, München, Germany; 3 Department of Neurology, University Medical Center of the Johannes Gutenberg-University Mainz, 55131 Mainz, Germany; 4 Centre Commun de Microscopie Appliquée, Université Nice Sophia Antipolis, Nice, France; 5 Department of Neurology, University of Münster, Albert-Schweizer Campus 1, 48149 Münster, Germany

## Abstract

Recombinant TWIK2 channels produce weak basal background K^+^ currents. Current amplitudes depend on the animal species the channels have been isolated from and on the heterologous system used for their re-expression. Here we show that this variability is due to a unique cellular trafficking. We identified three different sequence signals responsible for the preferential expression of TWIK2 in the Lamp1-positive lysosomal compartment. Sequential inactivation of tyrosine-based (Y_308_ASIP) and di-leucine-like (E_266_LILL and D_282_EDDQVDIL) trafficking motifs progressively abolishes the targeting of TWIK2 to lysosomes, and promotes its functional relocation at the plasma membrane. In addition, TWIK2 contains two N-glycosylation sites (N_79_AS and N_85_AS) on its luminal side, and glycosylation is necessary for expression in lysosomes. As shown by electrophysiology and electron microscopy, TWIK2 produces functional background K^+^ currents in the endolysosomes, and its expression affects the number and mean size of the lysosomes. These results show that TWIK2 is expressed in lysosomes, further expanding the registry of ion channels expressed in these organelles.

## Introduction

In mammals, the family of two-pore domain potassium channels (K_2P_) comprises 15 members^1–3^. These channels are dimers of subunits containing two pore domains (P1 and P2), four membrane-spanning helices (M1–M4) and a large extracellular M1P1 loop^4, 5^. They produce leak K^+^ conductances, maintaining the resting membrane potential and counteracting membrane depolarization and cell excitability. K_2P_ channels are tightly regulated by different physiological agents and stimuli, including pH, temperature, stretch and bioactive lipids. They are implicated in many physiological and pathological roles, as diverse as cell volume regulation, hormone secretion, apoptosis, depression, breathing control, perception of pain and control of blood flow (for recent reviews see refs 6, 7).

The levels of currents produced by these channels are not only controlled by regulation of their activity at the plasma membrane, but also indirectly *via* controlled biogenesis, intracellular sorting and trafficking^8^. Among the K_2P_ channel family, 4 of them were considered as “silent” because of their inability to produce measurable currents in heterologous expression systems: TWIK1 (KCNK1, K_2P_1.1)^9^, THIK2 (KCNK12, K_2P_12.1)^10, 11^, KCNK7 (K_2P_7.1)^12^ and TASK5 (KCNK15, K_2P_15.1)^13, 14^. Two of them, TWIK1 and THIK2, have been further investigated and their apparent “silence” was related to intracellular retention as well as low intrinsic channel activity when they are present at the plasma membrane^9, 15–19^. THIK2 and KCNK7 are mainly present in the endoplasmic reticulum whereas TWIK1 is expressed in recycling endosomes. For TWIK2, the original studies reported various levels of current expression depending on the animal species in which TWIK2 was cloned, and on the heterologous systems used to express the recombinant channels^20–23^. The discrepancies varied broadly from no current to measurable currents.

TWIK2 is expressed in several mammalian tissues including secondary lymphoid organs and the vascular system^20–23^. Its expression in the central nervous system is very weak^3^. TWIK2 knock-out mice exhibit an increased mean arterial and pulmonary blood pressure, depolarized resting membrane potential in freshly dispersed smooth muscle cells and increased vascular contractility^24, 25^. In this study, we studied the mechanism of its intracellular distribution in transfected cells.

## Results

### Expression of TWIK2 protein and current in lysosomes

**Figure 1:**
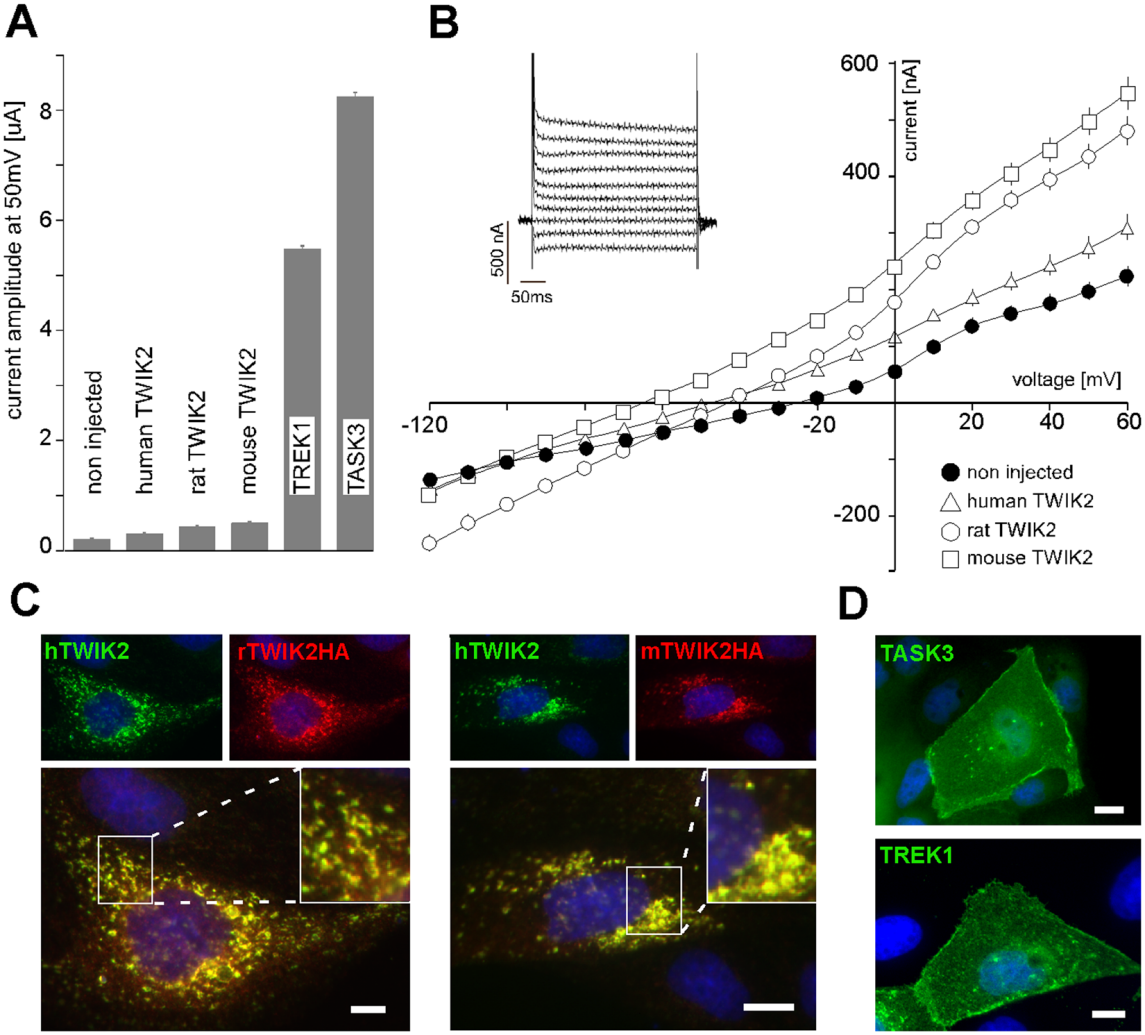
Comparison of human, rat and mouse TWIK2 channels in heterologous expression systems. TWIK2 currents were recorded in *Xenopus* oocytes 24 h after cRNA injection. (**A**) Current amplitude at +50 mV of human, rat and mouse TWIK2 compared to human K_2P_ channels TREK1 and TASK3. TWIK2 current is small (<1 µA) but significantly different from non-injected cells (non injected n = 56, human TWIK2 n = 23, p < 0.001, rat TWIK2 n = 47, p < 0.001, mouse TWIK2 n = 33, p < 0.001). Each value is the mean ± sem, statistic evaluation was done using Mann-Whitney test. (**B**) I-V relationships deduced from recordings with voltage pulses ranging from −120 mV to +60 mV in 10 mV steps from a holding potential of −80 mV (non injected n = 56, human n = 23, rat n = 47, mouse TWIK2 n = 33). The “inset” represents the evoked current shape of mouse TWIK2, 20 mV steps. (**C**) Human and rodent TWIK2 co-localization in transfected MDCK cells. Human TWIK2 was stained with rabbit polyclonal TWIK2 antibody and rat and mouse TWIK2HA with mouse monoclonal HA antibody. Overlap of green and red fluorescence appears in yellow. (**C**) TASK3 (in green) and TREK1 (in green) in transfected MDCK cells.

Recombinant mouse, rat and human TWIK2s were expressed in *Xenopus* oocytes. We could record weak but significant K^+^ currents (<1 µA at +50 mV, Fig. 1A). These currents are rapidly inactivating as previously shown for human TWIK2 in transfected mammalian cells (Fig. 1B)^20–23^. However, they are much smaller than the currents usually produced by other K_2P_ channels that are expressed at the plasma membrane. Under the same conditions TREK1 (KCNK2, K_2P_2.1) and TASK3 (KCNK9, K_2P_9.1) channels would produce more than 5 µA of current, suggesting that TWIK2 is mainly expressed in an intracellular compartment as shown previously for “silent” TWIK1^19^ and THIK2^18^. To study its cellular localization, we expressed TWIK2 into MDCK cells, an epithelial cell line classically used to study trafficking of membrane proteins^8^. Following immunolabeling of TWIK2, these cells display an intracellular staining (Fig. 1C). Human TWIK2 was labeled using a rabbit polyclonal TWIK2 antibody that does not cross-react with rodent channels^22^, whereas rat and mouse TWIK2s were labeled using HA-antibodies directed against a HA-tag fused to their C-terminus (C-ter). Figure 1C shows the co-localization of human TWIK2 co-expressed with rat or mouse TWIK2s, demonstrating that all three isoforms share the same intracellular distribution. A previous study has shown a relocation of rat TWIK2 from an intracellular localization to the plasma membrane after 72 h of expression in CHO cells^21^ but we could not confirm this observation in MDCK cells (Suppl. Fig. 1).

**Figure 2:**
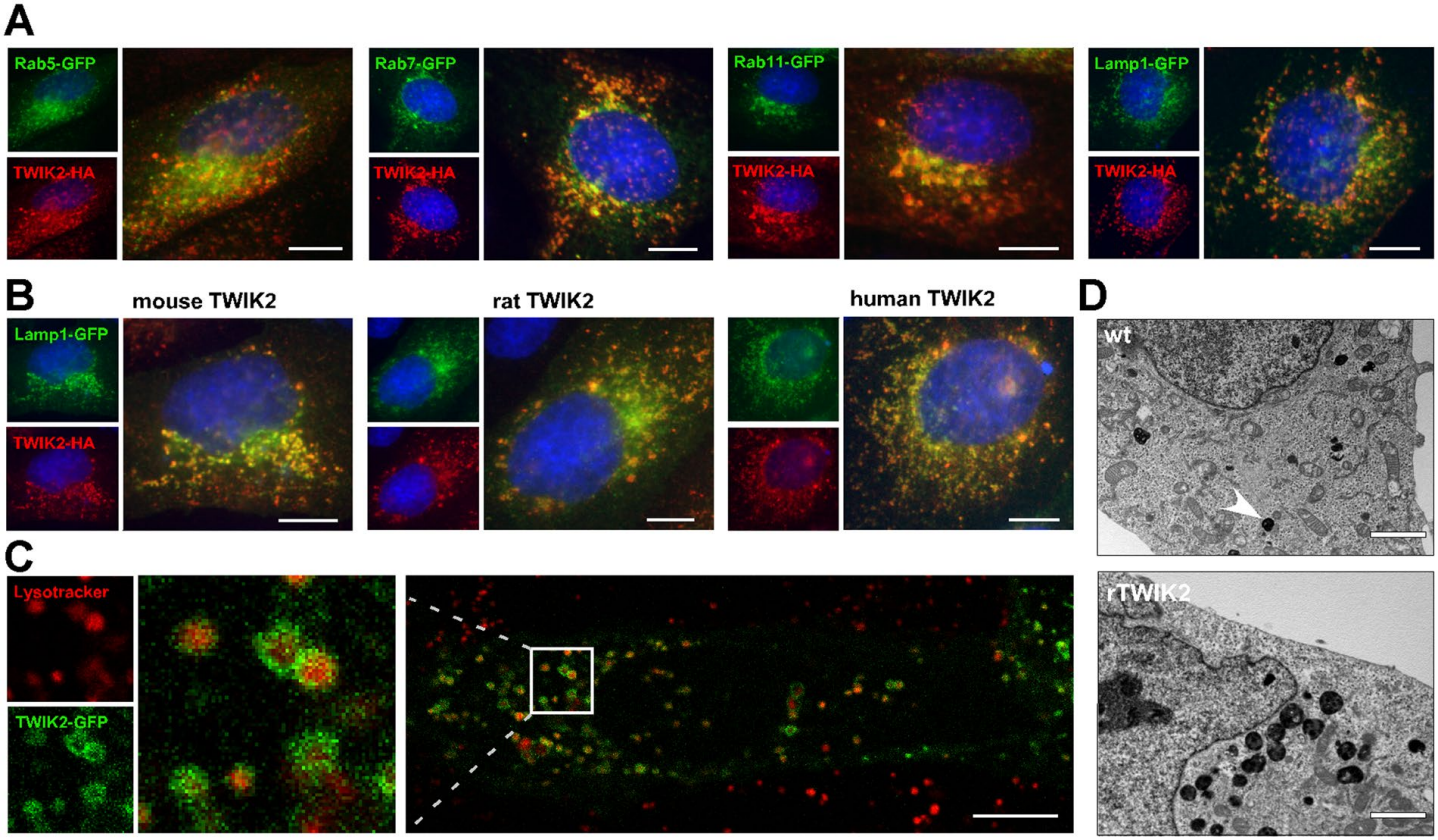
Localization of TWIK2 in lysosomal membranes. Representative images of MDCK cells expressing (**A**) GFP-coupled compartment markers and rat TWIK2HA, and (**B**) Lamp1-GFP together with mouse, rat or human TWIK2HA. TWIK2 was stained with HA antibody (red) and nuclei were visualized with Hoechst33342 (blue). Scale bar: 10 µm. (**C**) Image of a living MDCK cell expressing rat TWIK2-GFP and incubated with lysotracker (red), staining the lysosomal lumen. Scale bar: 10 µm. (**D**) Electronic microscopy images of a cell expressing rat TWIK2 (lower panel) or of a control cell (upper panel). Scale bar: 2 µm. Lysosomes are dense bodies (plain arrow).

The cellular localization of TWIK2 was further studied by co-expressing rat TWIK2 with different intracellular proteins coupled to GFP. A partial co-localization of TWIK2 with Rab7-GFP^26^ and Lamp1-GFP^27^ was observed, suggesting expression of TWIK2 in late endosomes and lysosomes (Fig. 2A). Labeling endogenous Lamp1 using a specific antibody gave the same result confirming the localization of rat, mouse and human TWIK2 in lysosomes (Fig. 2B). Next we analyzed the distribution of a rat TWIK2-GFP fusion protein in living cells. Staining lysosomes with Lysotracker enabled us to observe TWIK2 in the lysosomal membranes (Fig. 2C). Live video microscopy shows the movement of these lysosomes (Suppl. Video). TWIK2 expression did not seem to change mobility of lysosomes but affects their number and mean size (Fig. 2D). Cells expressing TWIK2 have nearly 2.5 times more lysosomes than WT cells (37.00 ± 3.91 lysosomes per section plane *vs* 13.41 ± 2.15, n = 20, p < 0.01, Student’s non-paired t test). Lysosomes of TWIK2-expressing cells are nearly 3 times larger than lysosomes in control cells (0.29 ± 0.02 µm^2^ *vs* 0.12 ± 0.01, n = 20, p < 0.01, Student’s non-paired t test) (Fig. 2D).

**Figure 3:**
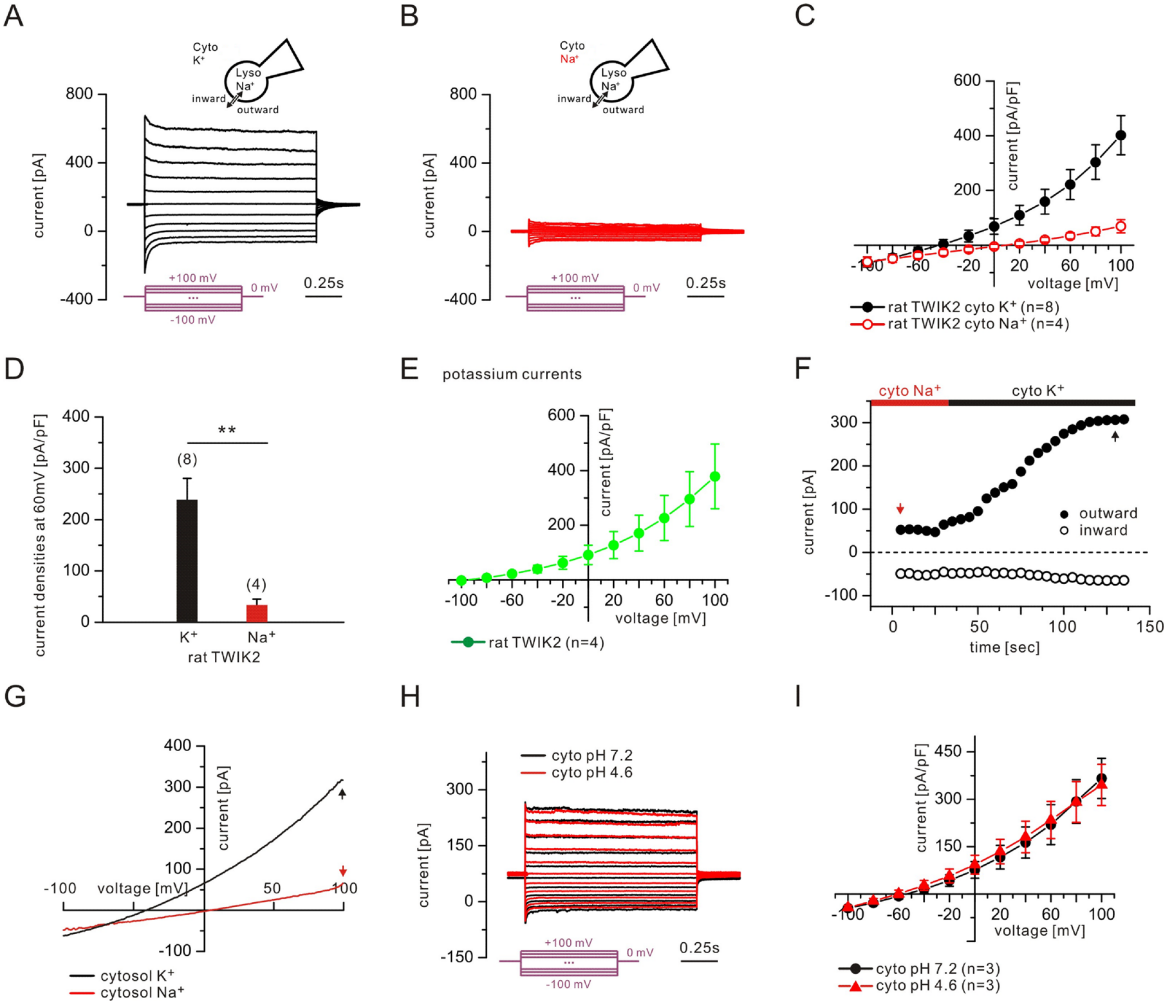
TWIK2 currents in endolysosomes. Endolysosomal currents were recorded from HEK293T cells expressing rat TWIK2. **(A)** Currents recorded using a high K^+^-containing bath solution (145 mM K^+^, pH 7.2). Step protocols from −100 to +100 mV with a 20 mV step were applied from a holding potential of 0 mV. **(B)** Currents recorded using a high Na^+^-containing bath solution (145 mM Na^+^, pH 7.2). Same step protocol as in (**A**). **(C)** I-V relationships constructed from the currents recorded in (**A**) and (**B**). **(D)** Averaged current densities at 60 mV. **(E)** Potassium I-V curves (defined as ${I}_{{\rm{cyto}}{\rm{high}}{{\rm{K}}}^{+}}-{I}_{{\rm{cyto}}{\rm{high}}{{\rm{Na}}}^{+}}$). **(F)** Representative continuous recordings using ramp protocols (−100 mV to +100 mV in 500 ms, every 5 s, from a holding potential of 0 mV). **(G)** Recordings at time points (black and red arrows) in **(F)** were used for the I-V relationships. **(H)** Current recorded at cytosolic pH 7.4 and pH 4.6. Outward current denotes flow of positive charge into the lumen (pipette) from the cytosol (bath). Numbers of patched endolysosomes are shown in parentheses. Data are represented as mean ± s.e.m. Same step protocol as in (**A**). **(I)** I-V relationships constructed from the currents recorded in (**H**).

To record the lysosomal K^+^ permeability of TWIK2, we used a modified conventional whole-endolysosome manual patch-clamp technique. Transfection of TWIK2 generated major K^+^ outward currents (from cytosol to endolysosomal lumen) not observed in control cells (Fig. 3 and Suppl. Fig. 2). When recorded with symmetrical Na^+^ solutions, outward cation currents were reduced in endolysosomes of HEK293T cells transfected with TWIK2 (Fig. 3A–G), showing that TWIK2 formed a K^+^ channel on endolysosomal membranes. The current-voltage (I-Ψ) relationship showed an outward rectification under high cytosolic K^+^ condition (high K^+^ gradient) as previously demonstrated^22^. It has also been proposed that TWIK2 currents were not sensitive to extracellular pH (similar to endolysosomal luminal) but slightly reduced by intracellular acidification induced by application of extracellular 2,4-dinitrophenol or carbon dioxide^20^. However, under our endolysosomal recording conditions, cytosolic acidification has no obvious effects on the amplitudes or I-Ψ relationship (Fig. 3H,I). We further confirmed that TWIK2 was not affected by direct intracellular acidification by recording a mutant form of TWIK2 predominantly found at the plasma membrane (TWIK2 Y_308_A IL_289/290_AA, described in the next paragraph). As expected, TWIK2 currents recorded in endolysosomes (Fig. 3A,C) and at the plasma membrane (Suppl. Fig. 3) have the same overall properties, i. e. an inactivating component, opening at all potentials and a marked resistance to cytosolic acidification.

### Signal sequences in the C-ter of TWIK2 control its trafficking/transport to lysosomes

**Figure 4:**
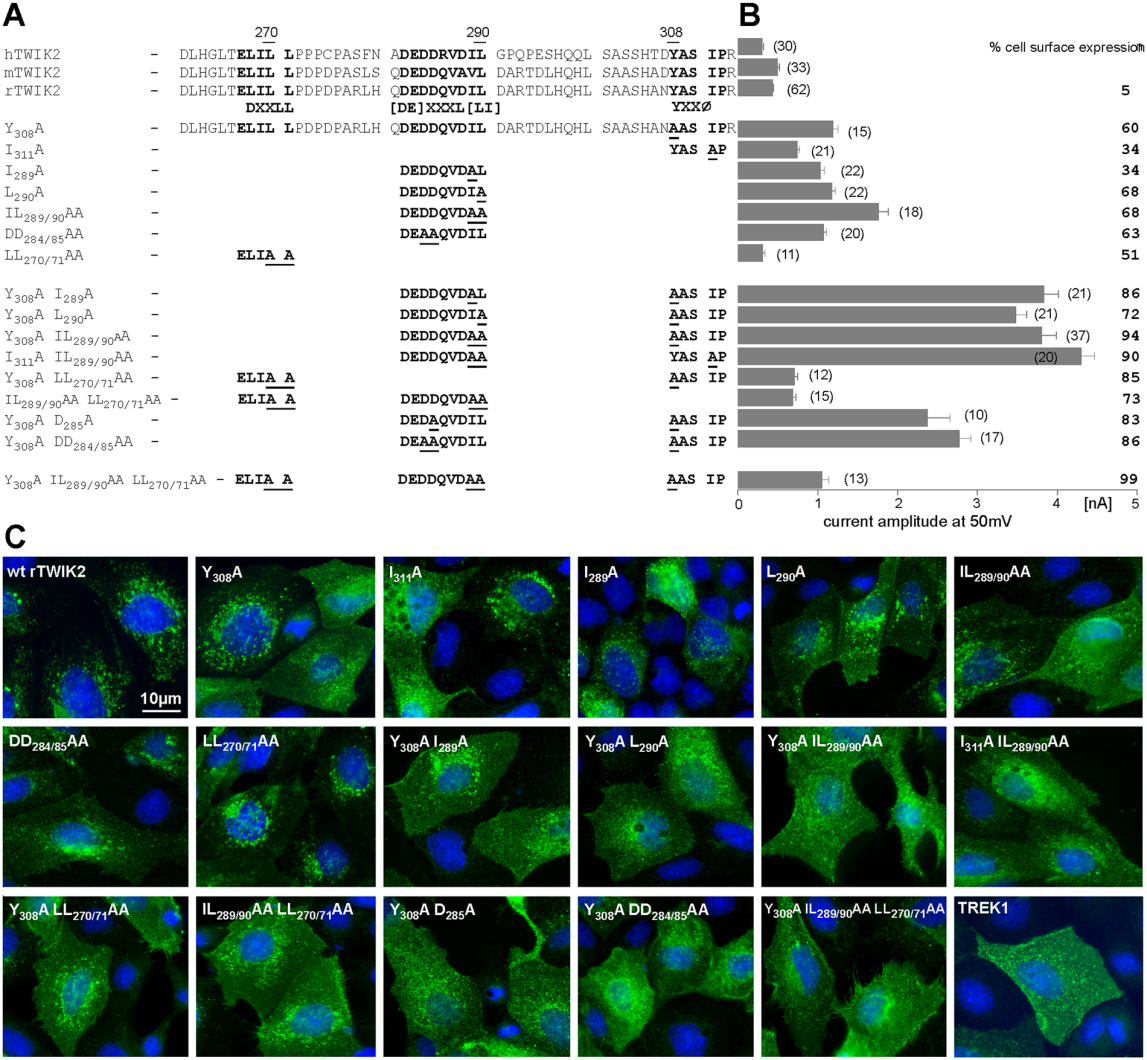
Identification of trafficking motifs in the C-ter of TWIK2. (**A**) Sequence of human, rat and mouse TWIK2 C-ter. The trafficking motifs are shown in bold. Overview of the set of generated mutants, in which underlined amino acids were replaced by an alanine residue. (**B**) Current amplitudes at +50 mV of the corresponding rat TWIK2 mutants recorded in *Xenopus* oocytes (number of recorded oocytes) expressed as mean current amplitude ± sem. Mutant rat TWIK2HA channels were transfected into MDCK cells and stained with HA antibody. % cell surface expression corresponds to the percentage of transfected cells showing a clear expression of TWIK2 at the plasma membrane. (**C**) Representative images of MDCK cells expressing the different TWIK2 mutations shown in (**B**) and TREK1. Scale bar: 10 µm.

Sorting of transmembrane proteins to intracellular organelles is mediated by trafficking signals embedded in their cytosolic domains, which are recognized by elements of the cell machinery such as adaptor protein (AP) complexes. The C-ter region following the last transmembrane helix (M4) constitutes the longest cytoplasmic sequence of TWIK2. Despite its variability, it contains potential trafficking motifs that are present in all mammalian sequences, and partially conserved in *Xenopus tropicalis* and *Danio rerio* (Suppl. Fig. 4). To check the functional relevance of these motifs, we progressively deleted the C-ter of rat TWIK2 (Suppl. Fig. 5A). However, all truncated channels displayed an intracellular localization in transfected MDCK cells (Suppl. Fig. 5B). We next introduced point mutations in the three potential signal sequences. Figure 4A displays the complete set of rat TWIK2 mutants. These mutants were either expressed in *Xenopus* oocytes to compare current amplitudes (Fig. 4B), or transfected and immunolabelled in MDCK cells to study cellular distribution (Fig. 4C). The first potential motif is a tyrosine-based motif YXXØ at the very end of the C-ter (YASIP in mammals, Y[NS]SIN in toad and zebrafish)^28^. In TWIK2, replacing tyrosine 308 (Y_308_A) or isoleucine 311 (I_311_A) with an alanine promoted a significant increase of cell surface expression in MDCK cells, and a 2 to 3-fold current increase in oocytes (1.19 ± 0.06 µA for TWIK2 Y_308_A and 0.74 ± 0.03 µA for TWIK2 I_311_A *versus* 0.43 ± 0.02 µA for TWIK2 at 50 mV). The second potential signal sequence is related to a di-leucine-type motif [DE]XXXL[LI] that is also present in TWIK1 and responsible for its constitutive endocytosis^9^. This motif (DEDDQVDIL in rat TWIK2) contains an acidic cluster of amino acids, which may also favor channel sorting to lysosomes. Alanine substitution of isoleucine 289 (TWIK2 I_289_A) and/or leucine 290 (TWIK2 L_290_A), as well as glutamic acids 284 and 285 of the acidic cluster (TWIK2 DD_284/285_AA) increased the expression of TWIK2 at the plasma membrane (Fig. 4B,C). Mutations of the tyrosine and di-leucine-like motifs show additive effects leading to a 9 to 10- fold current increase (3.8 ± 0.2 µA for TWIK2 Y_308_A IL_289/290_AA, 4.3 ± 0.2 µA for TWIK2 I_311_A IL_289/290_AA *versus* 0.43 ± 0.02 µA for TWIK2 at 50 mV) and to a strong cell surface relocation (Fig. 4B,C). Finally, the third motif belongs to the DXXLL type (ELILL in mammalian TWIK2s), which is known to promote sorting directly from the Trans-Golgi-Network (TGN) to endosomes without trafficking *via* the plasma membrane^28^. Alanine replacement of leucines 270 and 271 (TWIK2 LL_270/271_AA) favors cell surface expression (Fig. 4B,C), but with less current expression when compared to the wild type channel (0.31 ± 0.03 µA for TWIK2 LL_270/271_AA *versus* 0.43 ± 0.02 µA for TWIK2 at 50 mV). The same inhibitory effect is observed when this mutation is combined with mutations of the two other trafficking motifs, even if the mutated channel is fully expressed at the cell surface with no intracellular labeling. These results suggest that the DXXLL motif is an active trafficking signal but that mutations of this motif affect TWIK2 channel activity, probably because of its vicinity with the last membrane-spanning segment M4 that contributes to the internal mouth of the ionic pore.

**Figure 5:**
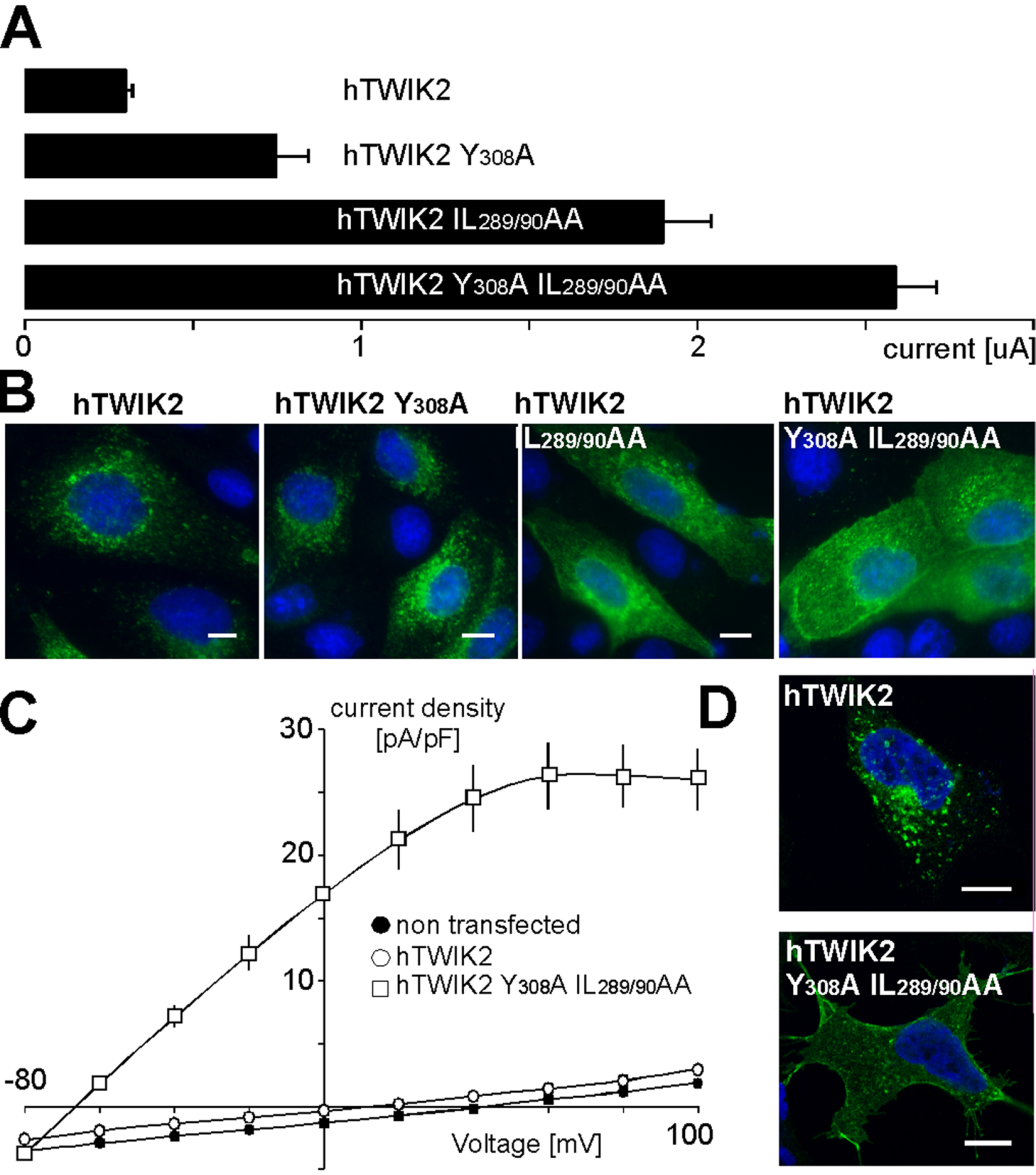
Human TWIK2 shows the same properties as rat TWIK2 when expressed in heterologous expression systems. Human wild type and mutated TWIK2 channels were expressed as indicated in (**A**) *Xenopus* oocytes (current amplitudes at +50 mV, (number of recorded oocytes)), (**B**) MDCK cells for immunodetection with anti-HA antibody, (**C**) HEK cells for electrophysiological recordings (n = 15), and (**D**) for immunodetection with anti-HA antibody.

The results presented so far were obtained using rat TWIK2. By using the same approach, we next confirmed the importance and conservation of tyrosine and di-leucine-like motifs in human TWIK2. Inactivation of these motifs resulted in more expression at the plasma membrane in MDCK cells and higher current amplitudes in oocytes (0.75 ± 0.09 µA for TWIK2 Y_308_A, 1.90 ± 0.14 µA for TWIK2 IL_289/290_AA and 2.59 ± 0,12 µA for TWIK2 Y_308_A IL_289/290_AA *versus* 0.30 ± 0.02 µA for TWIK2; Fig. 5A,B). We also expressed human wild type and mutated TWIK2 channels in HEK293 cells to carry out immunocytochemistry and electrophysiology in human cells (Fig. 5C,D). TWIK2 Y_308_A IL_289/290_AA produced 18 times more current than TWIK2 (26 ± 2.7 pA/pF, n = 10 at + 60 mV *versus* 1.4 ± 0.4 pA/pF n = 9; p < 0.001, Student’s non-paired t test). As expected more channels were detected by immunocytochemistry at the plasma membrane (Fig. 5D).

**Figure 6:**
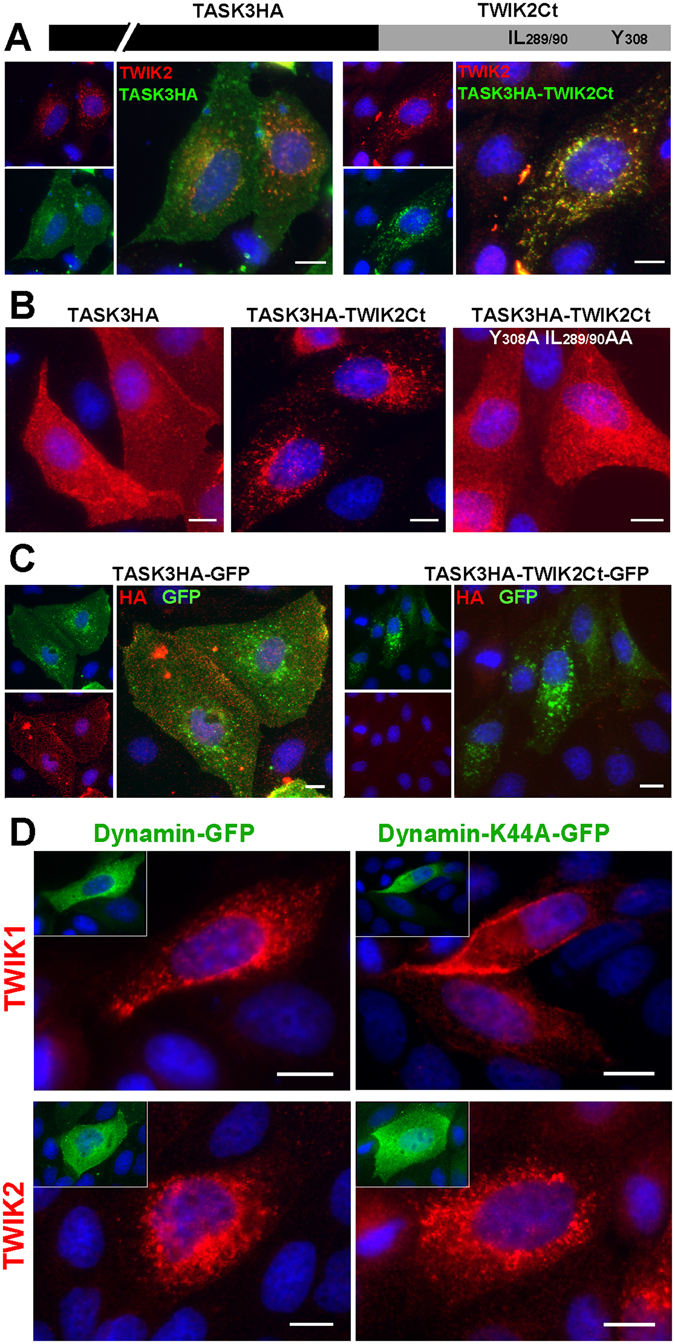
Cytoplasmic TWIK2 C-ter drives channel targeting to lysosomal membranes. (**A**) Co-transfection of rat TWIK2 with TASK3HA containing an extracellular HA-tag or TASK3HA-TW2Ct and staining with anti-HA (green) and anti-TWIK2 (red) antibody. (**B**) MDCK cell transfection with TASK3HA, TASK3HA-TW2Ct or TASK3HA-TW2Ct mutated at Y308A and IL289/290AA. (**C**) MDCK cell transfection with TASK3HA or TASK3HA-TW2Ct fused to GFP and stained without permeabilisation. (**D**) Co-expression of mouse TWIK1 or rat TWIK2 with Dynamin-GFP or Dynamin-K44A-GFP. Scale bar: 10 µm.

Together these results identify three trafficking motifs in the C-ter of TWIK2 that are responsible for its distribution to lysosomes. Are these signals sufficient to drive the relocation of a membrane protein from the plasma membrane to lysosomes? To address this question, we used a version of the TASK3 channel^29^ containing a HA-tag on its extracellular side (TASK3HA, Fig. 6A). We designed a chimeric channel by exchanging the cytoplasmic C-ter of TASK3HA with the corresponding C-ter of rat TWIK2 in TASK3HA (chimera noted TASK3HA-TW2Ct). TASK3HA and TASK3HA-TW2Ct were co-transfected with TWIK2 into MDCK cells, and their localization was analyzed using anti-HA and anti-TWIK2 antibodies. TASK3HA is located at the plasma membrane, whereas TASK3HA-TW2Ct co-localizes with TWIK2 in lysosomes (Fig. 6A). Mutating the tyrosine and di-leucine-like motifs in TASK3HA-TW2Ct promotes a re-expression of the chimeric channel at the plasma membrane (Fig. 6B) further confirming the role of these motifs, and demonstrating that the C-ter of TWIK2 is necessary and sufficient to promote membrane protein targeting to lysosomes. To visualize channel proteins without antibody staining, they were fused to GFP. When the cells are not permeabilized only TASK3HA-GFP, but not TASK3HA-TW2Ct-GFP, can be detected using HA antibody (Fig. 6C). This result suggests that either TWIK2 channels do not transit *via* the plasma membrane, or that the amounts present at the cell surface at any given time are not enough to enable detection. TWIK1 endocytosis is a dynamin-dependent mechanism^9^. Co-transfection of TWIK2 with a dominant-negative dynamin I (dynamin K_44_A) did not change channel distribution, suggesting a dynamin-independent trafficking mechanism (Fig. 6D).

**Figure 7:**
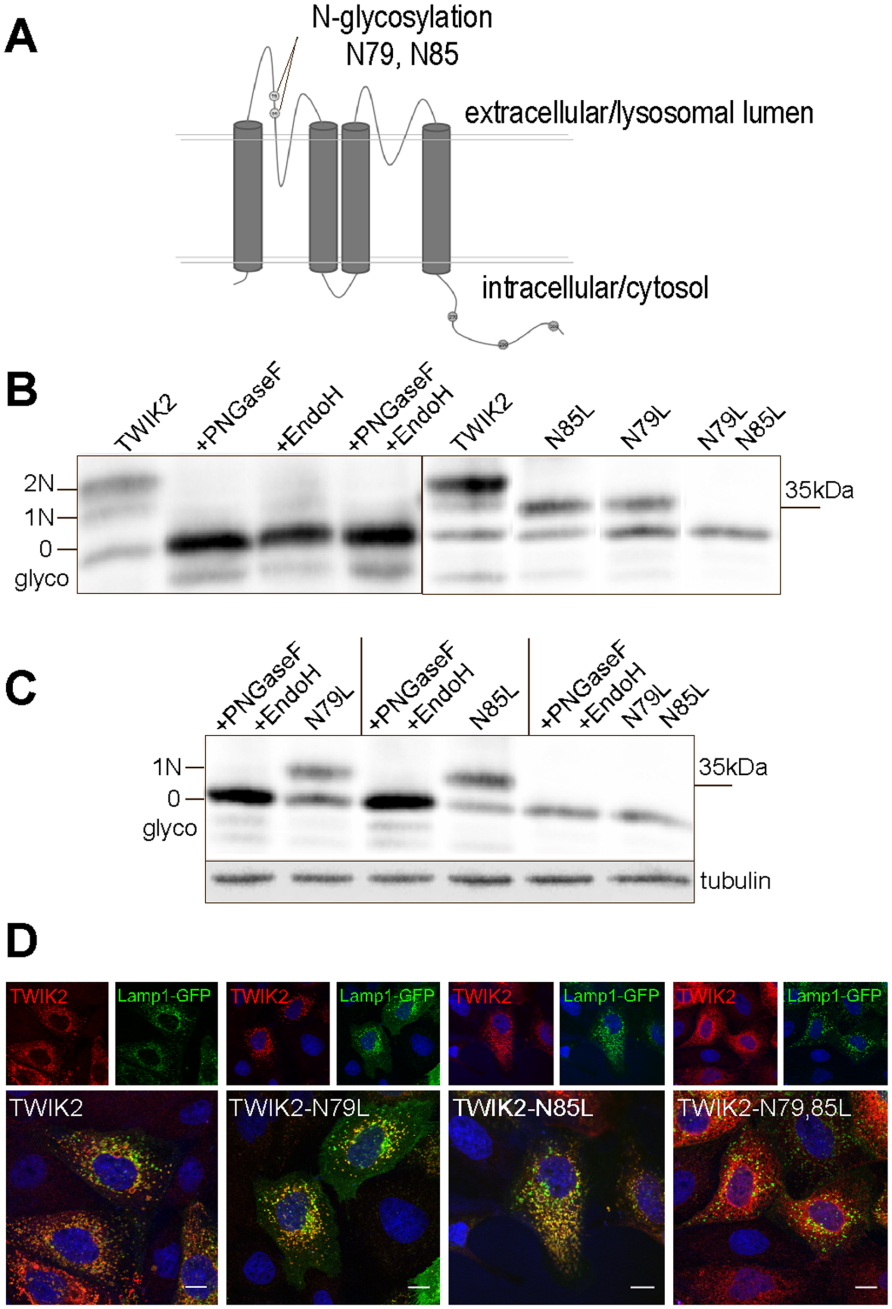
Glycosylation of rat TWIK2 in the M1-P1 extracellular/lumenal loop. (**A**) TWIK2 membrane topology and localization of the N-glycosylation sites. (**B**) TWIK2 deglycosylation. Protein lysates from COS cells expressing TWIK2 were incubated with PNGaseF and/or EndoH. The blot images have been cropped, only bands corresponding to glycosylated and deglycosylated TWIK2 isoforms are shown. (**C**) Mutation of the glycosylation sites (N to L) has the same effect as incubation with PNGaseF and EndoH. The blot images have been cropped, only bands corresponding to glycosylated and deglycosylated TWIK2 isoforms are shown. (**D**) Immunolabelling in MDCK transfected cells. TWIK2 N_79_L and TWIK2 N_85_L have a lysosomal distribution whereas TWIK2 N_79_LN_85_L is detected in the endoplasmic reticulum. Scale bar: 10 µm.

TWIK1 contains a functional N-glycosylation site on its extracellular side^5^. In TWIK2, asparagines 79 and 85 form potential N-glycosylation sites (N_79_AS and N_85_AS) that fit the consensus motif NXS/T (Fig. 7). To test glycosylation, protein lysates prepared from cells expressing rat TWIK2 were incubated with Endo-glycosidases EndoH and/ or PNGaseF, and analyzed by Western blot (Fig. 7B left panel). Three different bands were detected in the untreated lysates, but only the lower band was detected in the lysates treated with glycosidases. This shows that TWIK2 is glycosylated, and that the two upper bands correspond to glycosylated proteins. To test the role of the potential glycosylation sites N79 and N85, these asparagines were substituted by a leucine to create single (TWIK2 N_79_L and TWIK2 N_85_L) and double (TWIK2 N_79_L,N_85_L) mutants. The apparent molecular weights (MWs) of mutated TWIK2s are identical to the MWs of the two different glycosylated forms (Fig. 7B, right panel). Incubation of each single mutant with Endo-glycosidases leads to the deglycosylation of the other asparagine, with no gel shift for the double mutant. These results show that both N79 and N85 are glycosylated in TWIK2 (Fig. 7C). To study the effect of the mutations on TWIK2 cellular distribution, the mutated channels were transfected into MDCK cells and immunolabeled (Fig. 7D). Single mutants TWIK2 N_79_L and TWIK2 N_85_L display the same intracellular pattern as TWIK2, whereas double mutant TWIK2 N_79_L,N_85_L shows a strong ER-like staining, suggesting that TWIK2 needs to be glycosylated on at least one asparagine to be stably expressed in lysosomes.

## Discussion

Our results show for the first time that recombinant TWIK2 is located in lysosomes, and that this distribution depends on its cytoplasmic C-ter region. When fused to TASK3, a channel that distributes at the plasma membrane, this domain is sufficient to drive the targeting of the chimeric channel to lysosomes. The TWIK2 C-ter region contains three active trafficking motifs^28^. The first sorting signal is located at the very end of its C-ter, and is a tyrosine-based signal that conforms to the YXXØ consensus motif. This type of motif is essential for rapid internalization from the plasma membrane, but is also implicated in the targeting of proteins to lysosomes, for example in the targeting of Lamp1^30–32^. Lysosomal-sorting YXXØ motifs usually have a glycine residue preceding the critical tyrosine residue and acidic residues at the X positions, but this is not the case for TWIK2. The position of the motif within the cytosolic domain of the membrane protein is another important characteristic determining its endocytic or lysosomal-targeting role. Purely endocytic YXXØ signals are not situated at the C-ter of the proteins, but 10–40 residues next to a transmembrane domain. In contrast, the YXXØ motif in the TWIK2 sequence is located at the extremity of its C-ter with only two residues downstream, as observed in other proteins targeted to late endosomal-lysosomal compartments, including Lamp1^32^. The second signal sequence of TWIK2 is related to the [DE]XXXL[LI]-type. This di-leucine-based motif mediates rapid internalization and targeting to endosomal-lysosomal compartments, suggesting that they are active both at the plasma membrane and in intracellular locations^33, 34^. Such a motif is present in TWIK1 and is responsible for its rapid and constitutive endocytosis^9^. Similar to TWIK1, the TWIK2 sequence bears a cluster of acidic amino acids preceding this signal that strengthens its sorting to lysosomes. Both YXXØ- and [DE]XXXL[LI]-type motifs bind to adaptor proteins (AP-1, −2 and −3), and membrane proteins containing these motifs are transported to endosomal-lysosomal compartments^35–37^. Interestingly, in TWIK2 these two motifs have synergistic effects suggesting that they do not bind at the same site in AP complexes. The third signal sequence of TWIK2 belongs to the recently discovered DXXLL-type signal^28^. This other type of dileucine-based motif is responsible for cycling between TGN and endosomes^38–40^. In contrast to YXXØ- and [DE]XXXL[LI]-type motifs, DXXLL sequences do not bind to APs, but to Golgi-localized, γ-ear-containing, ADP-ribosylation factor-binding proteins (GGAs), which function like adaptor proteins^41^. For TWIK2, our results show that the three trafficking motifs have additional effects, and lead to a specific targeting to lysosomes. As they are vesicles specialized in protein degradation, lysosomal proteins need to be protected against enzymatic proteolysis. One known mechanism is glycosylation, as demonstrated for Lamp1 and P2X4 channels. Glycosylation protects these lysosomal channels from degradation^42, 43^. TWIK2 is N-glycosylated in the M1-P1 loop facing the lysosomal lumen. When both glycosylated asparagines are mutated, glycosylation of TWIK2 is prevented. The resulting unglycosylated channel was not detected in lysosomes but in ER, suggesting that the unglycosylated channel is not addressed to lysosomes, or is rapidly degraded in these organelles in the absence of the protection afforded by glycosylation.

Although still under investigation, diverse physiological roles have been suggested for silent K_2P_ channels. Ion channel selectivity of TWIK1 is dynamically regulated by acidic pH suggesting that its recycling between the plasma membrane and internal acidic stores may determine its inhibitory or excitatory influence on cell excitability^17, 44^. TWIK1 may also transport K^+^ and Na^+^ in endosomes and thus influences the biology of this recycling compartment. THIK2 is retained in the endoplasmic reticulum (ER), where its expression may affect calcium storage and release throughout its capacity to transport K^+^ counter-ions^8, 16, 18^. Here we show that TWIK2 is mainly present in lysosomal membranes of transfected cells. Its expression affects the mean size of the lysosomes suggesting an active role in these organelles. K^+^ plays a role in the electrical potential of endosomes and lysosomes, thereby regulating pH stability and organelle function^45^. Beside K^+^, there are other cations, Na^+^ and Ca^2+^, as well as the anion Cl^-^ that help to counterbalance the H^+^ pumping to generate and maintain the endo/lysosomal acidic pH. A number of channels involved in mediating the counter-ion permeability are known: CFTR for Cl^-^ conductance, TPC for Na^+^ and P2X4 and TRPM2/TRPMLs for Ca^2+^ ^45–49^. There was also evidence for a “leak-like” K^+^ current in lysosomal membranes^50^. A recent report describes TMEM175, a newly discovered K^+^ channel that produces this K^+^ current in lysosomes^51^. Unlike any of the other K^+^ channels cloned to date from viruses, bacteria, fungi, plants and animals, TMEM175 has no GYG sequence signature-pore domain. This very unusual structure as well as the absence of reports describing classical K^+^ channels in lysosomes suggested that TMEM175 is a highly specialized organelle channel tailored to the lysosomal function. We show here that TWIK2 is another, voltage-independent K^+^ channel expressed in lysosomal membranes. Both TMEM175 and TWIK2 have a wide tissue distribution suggesting that the two proteins may act together in regulating lysosomal function. However, expression levels of TWIK2 are more pronounced in spleen and PBL suggesting a specialized physiological role in these cells. In phagocyting immune cells like macrophages and antigen-presenting immune cells like dendritic cells, the endo-lysosomal compartment has specialized functions that are different from its general role in degradation and recycling of proteins and amino acids. These specialized vesicles take part in cell activation processes triggered by Ca^2+^-release into the cytosol^52^. They have a crucial function in antigen processing and presentation, as well as cell migration and exocytosis^53–55^. During starvation, lysosomes additionally play a role in the autophagocytosis^56^. TWIK2 expression in cells bearing specialized endo-lysosomal compartments may point to a particular role in these vesicles, in addition to the “basic” channel equipment.

In summary, this study provides the first evidence of a K_2P_ channel expressed in lysosomes, likely functioning as a lysosomal K^+^ channel in certain types of cells. Further studies using the knock-out mouse model of TWIK2 should help to decipher the subcellular localization of the native TWIK2 channels and their physiological role in lysosomes.

## Methods

### Antibodies and Reagents

Rabbit anti-TWIK2 polyclonal antibody has already been described^22^. Mouse anti-HA monoclonal antibody (clone HA.7) and rabbit anti-Lamp1 polyclonal antibody were purchased from Sigma-Aldrich (France). Lysotracker was from Life technologies (France). Cell nuclei were stained using Hoechst 33342 (ThermoFisher, USA).

### Molecular Biology

Mouse TWIK2 was cloned from lung tissue cDNA into pcDNA, pIRES-GFP and pLIN. Rat and human TWIK2 clones were kindly provided by Dr Amanda Patel (Valbonne, France). The HA-tag was added in frame at the 3′ end of the TWIK2 coding sequences by PCR. Point mutations, deletions and chimeras were obtained by PCR using standard procedures. All constructs were verified by sequencing. Dynamin-GFP and dynaminK_44_A-GFP were provided by Dr Christophe Lamaze (Paris, France). Rab5-, 7-, 11-GFP were from Dr Robert Lodge (Laval, Canada), and Lamp1-GFP from Dr Esteban C. Dell’Angelica (Los Angeles, USA).

### Cell culture and Transfection

Madin-Darby canine kidney (MDCK) cells were grown in Minimal Essential Medium (MEM, Invitrogen) and COS-7 cells in DMEM (Invitrogen) both supplemented with 10% FCS, 100 U/ml penicillin and 100 U/ml streptomycin in humidified incubators with 5% CO_2_ at 37 °C. For transient transfection, MDCK cells were transfected using Lipofectamine 2000 (Invitrogen) according to the manufacturer’s instructions and COS-7 cells were transfected with DEAE-Dextran method using 1 µg DNA per 35 mm culture dish.

### **Two-electrode** recording

cRNA synthesis and *Xenopus* oocyte injection, as well as oocyte electrophysiological recordings were performed as described previously^57^.

### Patch-clamp recording

HEK293 cells were plated on a 35-mm dishes with a density of 30,000 cells/dish, transfected 24 hours after plating (0.8 µg of DNA/dish) and used between 24 and 48 h after transfection. Dishes were placed in the patch-clamp chamber and continuously perfused with the control bath solution containing (in mM): 140 NaCl, 10 TEA-Cl, 5 KCl, 3 MgCl2, 1 CaCl2, 10 HEPES and adjusted to pH 7.4 with NaOH. The pipette solution contained (in mM): 155 KCl, 3 MgCl2, 5 EGTA, 10 HEPES and adjusted to pH 7.2 with KOH. All experiments were performed at room temperature (21–22 °C). K_2P_ currents were observed using RK400 patch clamp amplifier (Bio-Logic Science Instruments), low-pass filtered at 3 kHz and digitized at 10 kHz using a 12-bit analog-to-digital converter Digidata-1322 (Axon Instrument, Sunnyvale, CA, USA). For current visualization and stimulation protocol application, we used commercial software (Clampex 8.2). The patch pipettes were double-step-pulled from haematocrit-capillaries (Hirschmann Laborgeraete, Germany) using a vertical puller (PC-10, Narishige International, London, UK). Filled pipettes had resistances of 2–4 MΩ. Whole-cell patch-clamp configuration was obtained at a holding potential of −80 mV. A voltage step protocol was applied consisting of 900 ms depolarizing steps going from −80 mV to +100 mV in 20 mV increments. Current amplitudes were expressed as current densities (pA/pF) and results are presented as mean ± standard error of the mean (SEM).

### Endolysosome recording

Whole-endolysosome recordings were performed by modified conventional patch-clamp as previously described^58^. HEK293T cells transfected with pIRESGFPrTWIK2 were used to study TWIK2-expressing organelles. 24 hours after transfection, HEK293T cells were treated with 1 μM vacuolin-1 overnight. Currents were recorded using an EPC-10 patch-clamp amplifier and PatchMaster acquisition software (HEKA, Lambrecht/Pfalz, Germany). Data were digitized at 40 kHz and filtered at 2.8 kHz. Recording pipettes had a resistance of 5–10 MΩ. Liquid junction potential was corrected. Unless otherwise indicated in the figure legends, the pipette solution (corresponding to luminal endolysosomal solution) contained (in mM): 140 NaMSA, 5 KMSA, 2 CaMSA, 1 CaCl2, 10 HEPES and 10 MES, pH 4.6. Bath solution (corresponding to cytosolic solution) contained (in mM): 140 KMSA, 5 KOH, 4 NaCl, 0.39 CaCl2, 1 EGTA, and 20 HEPES, pH 7.2. For cytosolic low pH recordings, pH was adjusted with HCl. For high Na^+^ recordings, NaMSA was used to replace KMSA. pH of bath solution and pipette solution was adjusted with KOH and MSA, respectively. All recordings were obtained at 21–23 °C and were analyzed using PatchMaster and Origin 6.1 (OriginLab, Northampton, MA) software.

### Western blot analysis

Transfected COS cells were scraped and lysed in lysis buffer (100 mM NaCl, 40 mM KCl, 1 mM EDTA, 20 mM HEPES, 10% Glycerol, 1% Triton-X, with Complete Protease Inhibitor from Roche) for 1 h at 4 °C. Lysates were cleared by 10 min centrifugation at 20,000 g. For deglycosylation, solubilized proteins were incubated with 250 U EndoH and/or 10 U PngaseF (Promega, France) at 37 °C overnight. Before separating proteins on a 10% polyacrylamide gel and blotting onto PVDF membrane (Hybond-P, Amersham Bioscience), lysates were boiled 5 min at 99 °C with Laemmli buffer containing reducing ß-Mercaptoethanol. Blots were blocked with 5% milk/PBS for 1 h and incubated with anti-TWIK2 polyclonal antibody (1:200, Alomone, Jerusalem, Israel) at 4 °C overnight or with anti-β-Tubulin monoclonal antibody (1:1000, clone TUB 2.1, Sigma-Aldrich, France) 1 h at RT. Secondary horseradish peroxidase coupled antibodies were diluted 1:10.000 and incubated 1 h at RT before detection by enhanced chemiluminescence reaction (ECL, Amersham Biosciences).

### Immunocytochemistry

Cells were grown and transfected on glass coverlips. 24 h, 48 h, or 72 h post transfection, cells were fixed 10 min with 4% paraformaldehyde and blocked and permeabilized with PBS containing 0,1% Triton-X and 5% horse serum for 15 min. Cells were incubated 3 h with primary antibodies, then 1 h with fluorescence labeled secondary antibodies at RT and in the dark. Fluorescence images were acquired and analyzed using an Axioplan2 Imaging Microscope (Carl Zeiss) and the Metavue software.

### Live cell imaging

Cells were plated on 25 mm glass coverslips and transfected with rat TWIK2-GFP-encoding plasmid. 24 h post transfection, Lysotracker (Molecular Probes) was added in the medium (final concentration 20 nM), and after 10 min cells were observed on a Zeiss LSM7800 confocal microscope.

### Lysosome size

MDCK cells were transfected with empty pIRES2eGFP vector or pIRES2eGFP-rTWIK2. 24 h after transfection, cells were fixed in 1.6% glutaraldehyde (Sigma-Aldrich) in 0.1 M phosphate immediately after medium removal, respectively. Samples were rinsed with cacodylate buffer 0.1 M buffer and then post-fixed in osmium tetroxide (1% in cacodylate buffer 0.1 M) for 1 h. After rinsing with distilled water, they were then dehydrated through an increasing ethanol series and embedded in epoxy resin. Ultrathin sections (70 nm) were collected on Formvar-coated copper grids, stained with uranyl acetate and lead citrate and examined with a Jeol JEM 1400 transmission electron microscope equipped with a SIS MORADA camera. For each condition 10 to 15 fields were analyzed. Results are the mean ± SEM.

## Electronic supplementary material

Supplementary Information

Supplementary Information
