# Supplementary material for: Recombinant tandem of pore-domains in a Weakly Inward rectifying K+ channel 2 (TWIK2) forms active lysosomal channels
Source: Sci Rep. 2017 Apr 5;7:649. doi: 10.1038/s41598-017-00640-8 (PMC5428834; doi:10.1038/s41598-017-00640-8)
Supplement: Supplementary file 1 — Supplementary Information [file 41598_2017_640_MOESM1_ESM.pdf]

Recombinant tandem of pore-domains in a Weakly Inward rectifying K^+ channel 2 (TWIK2) forms active lysosomal channels

Nicole Bobak, Sylvain Feliciangeli, Cheng-Chang Chen, Ismail Ben Soussia, Stefan Bittner, Sophie Pagnotta, Tobias Ruck, Martin Biel, Christian Wahl-Schott, Christian Grimm, Sven G. Meuth, Florian Lesage

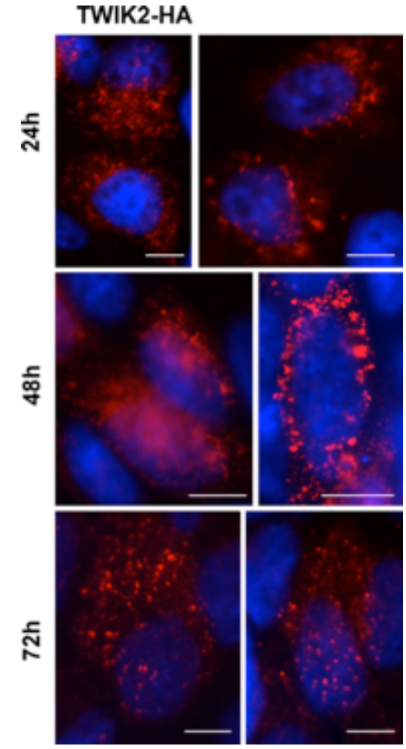

SUPPLEMENTAL FIGURE 1. ***TWIK2 expression stability during 72h in MDCK cells.*** TWIK2-HA was transfected into MDCK cells and stained with HA-antibody 24h, 48h and 72h post transfection. Scale bar 10µm.

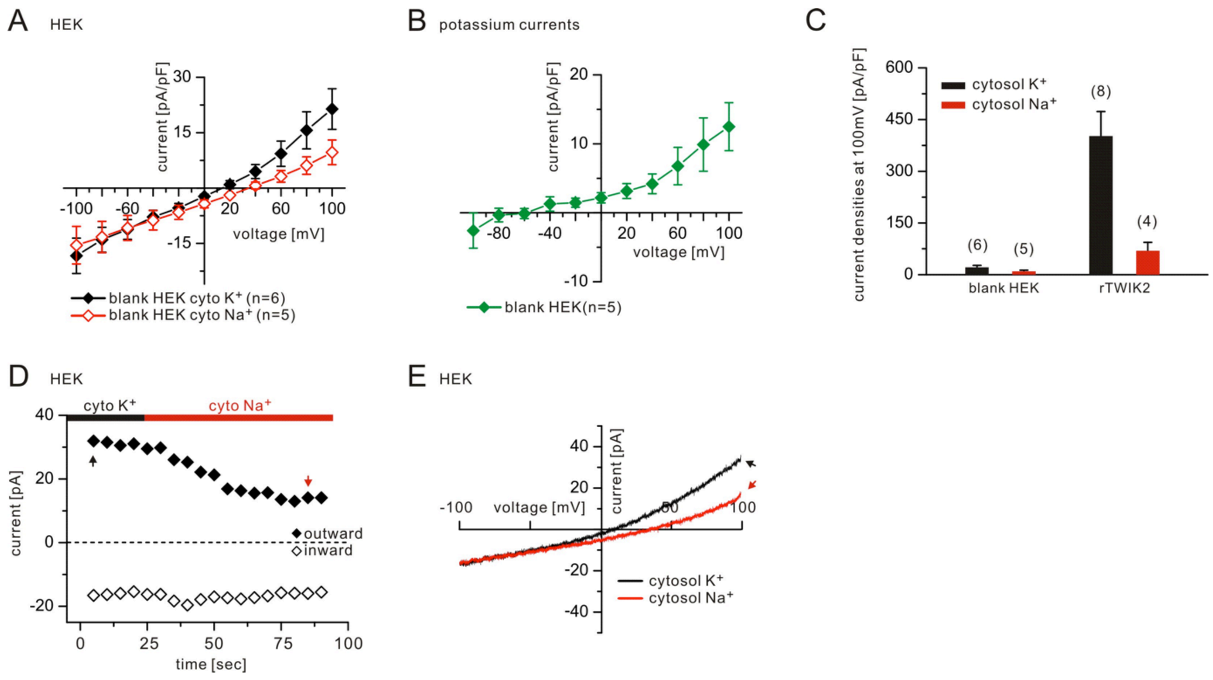

SUPPLEMENTAL FIGURE 2. ***Endolysosomal K^+ currents from non-transfected HEK293 cells.*** Cation currents were recorded in endolysosomes released from HEK293T cells. Representative currents were elicited by voltage step protocols from -100 to +100 mV (20 mV step) **(A and B)** or by voltage ramps from -100 to +100 mV (voltage holding at 0 mV) **(D and E)** with 145 mM K^+ and perfused with 145 mM Na^+ in the cytosol (bath) as indicated. Shown in **B** are potassium I-V curves ($I_{cyto\ high\ K^+}$ - $I_{cyto\ high\ Na^+}$) from blank HEK293T cells. **(C)** Column figures summarizing the current densities obtained at +100 mV. **(D)** Current amplitudes measured at -100 mV and +100 mV were used to plot the time course of activation. Data are represented as mean ± s.e.m.

The profile of these small endogenous K^+ currents is in agreement with previous data that suggest that other K^+ permeable ion channels are present on endolysosomal membranes such as TMEM175, TRPMLs and BK channels (Cang et al, 2015; Cao et al, 2015; Chen et al, 2014a).

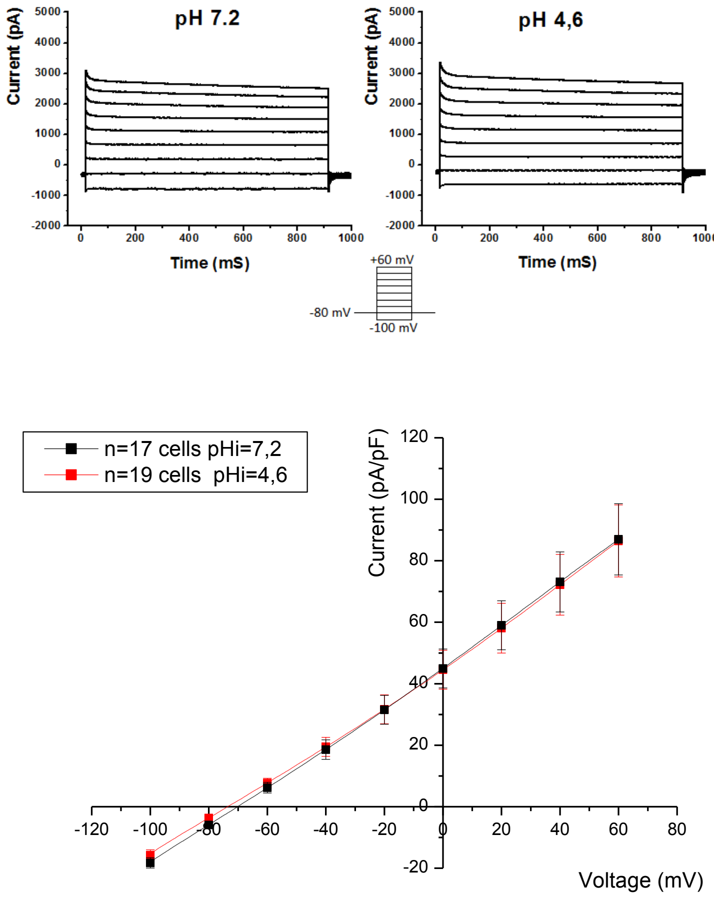

SUPPLEMENTAL FIGURE 3. ***Recordings of rat TWIK2 $Y_{308}A$ $IL_{289/290}AA$ at the plasma membrane of transfected HEK293T cells.***

H. Sapiens	-	DLHGLT**ELIL**	**L**PPPCPASFN	**A****DEDDRVDIL**	GPQPESHQQL	SASSHTD**YAS**	**IP**R
M. musculus	-	DLHGLT**ELIL**	**L**PDPDPASLS	Q**DEDDQVAVL**	DARTDLHQHL	SAASHAD**YAS**	**IP**R
R. norvegicus	-	DLHGLT**ELIL**	**L**PDPDPARLH	Q**DEDDQVDIL**	DARTDLHQHL	SAASHAN**YAS**	**IP**R
X. tropicalis	-	TDIFY	LPRLQ**DQDDQ**	**EPIL**ETTDYS	TRDLEPKRPL	ASESQPD**YSS**	**IN**R
D. rerio	-	HLPSC	**EEDEEDKEPI**	**I**EAGPEDDSP	EAEKASIKPL	DPSSQVS**YNS**	**IN**R

SUPPLEMENTAL FIGURE 4. ***Conserved trafficking motifs in the C-terminus of TWIK2.*** TWIK2-C-terminal-sequences of *Homo sapiens*, *Mus musculus*, *Rattus norvegicus*, *Xenopus tropicalis* and *Danio rerio*.

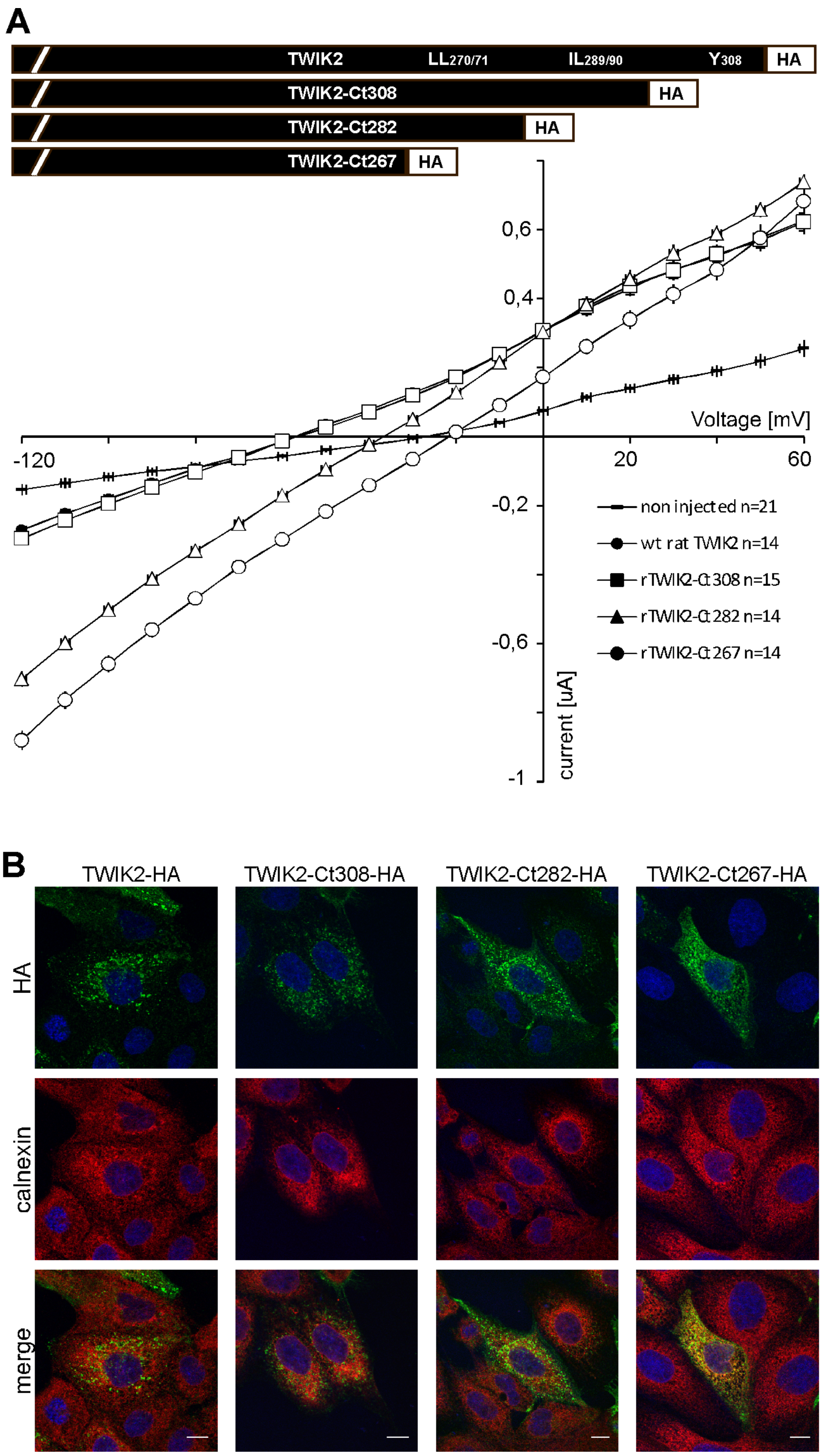

SUPPLEMENTAL FIGURE 5. ***Effect of the truncation of the TWIK2 C-ter on cell trafficking.*** (**A**) Truncated channels did not show significant changes in current amplitude when expressed and recorded in *Xenopus* oocytes and (**B**) showed intracellular localization when expressed and stained in MDCK cells. Scale bar: 10 μM.

SUPPLEMENTAL VIDEO

LEGEND OF THE VIDEO: Live HEK293 cells expressing TWIK2-GFP fusion protein (in green). Lysosmes are labelled with Lysotracker dye (in red).
